# Leaf Damage is Not the Answer! Reduced Herbivore Pressure Does Not Underpin Either Downhill or Uphill Range Shifts

**DOI:** 10.1002/ece3.73437

**Published:** 2026-04-06

**Authors:** Inna Osmolovsky, Zoe A. Xirocostas, Giancarlo M. Chiarenza, Eve Slavich, Angela T. Moles

**Affiliations:** ^1^ Evolution & Ecology Research Centre, School of Biological, Earth and Environmental Sciences UNSW Sydney Sydney New South Wales Australia; ^2^ School of Life Sciences, University of Technology Sydney Sydney New South Wales Australia; ^3^ ARC Training Centre for Healing Country, Department of Molecular and Life Sciences Curtin University Perth Western Australia Australia; ^4^ Stats Central, Mark Wainwright Analytical Centre UNSW Sydney Sydney New South Wales Australia

**Keywords:** climate change, downhill range shifts, herbivory, interaction opportunists hypothesis, leaf damage

## Abstract

Many organisms are observed shifting their ranges uphill or toward the poles in response to climate change. However, recent studies suggest that 20%–37% of species are exhibiting shifts in counterintuitive directions, that is, downhill or toward the equator. The Interaction Opportunists Hypothesis suggests that counterintuitive shifts might be induced by climate change‐driven decreases in enemy pressure at species' warm range edges. To test this hypothesis, we estimated the amount of leaf damage and the number of damage types in nine alpine plant species native to Kosciuszko National Park, Australia, that have shifted their ranges uphill, downhill, or remained stable. Contrary to our prediction, the amount of leaf damage and the number of damage types experienced at species' warm edge versus distribution core did not differ between downhill, uphill, and non‐shifting species. These results do not support the idea that reduced enemy pressure drives downhill range shifts. However, counterintuitive range shifts may be underpinned by changes in other biotic interactions, such as soil pathogen attacks, competition, or seed predation. Currently, it remains unclear why species are shifting their ranges toward warmer climates. Advancing understanding of counterintuitive shifts is critical for accurately predicting the likely impact of climate change on species, the composition and reorganisation of ecological communities, and ecosystem functioning into the future.

## Graphical abstract

While many plant species are shifting their ranges uphill in response to climate change, a substantial portion of species have exhibited a downhill shift, opposite to the expected response under climate change. We tested the hypothesis that these counterintuitive range shifts may be driven by a reduction in enemy (herbivores and pathogens) pressure. Surprisingly, we did not observe a reduction in herbivory at the expanding range of downhill shifting species. Our results suggest that other factors may be driving species to expand their ranges downhill.

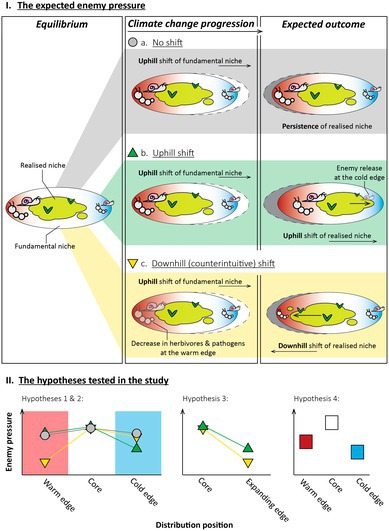

## 1 Introduction

Empirical evidence suggests that over half of species are shifting their ranges uphill or poleward in response to climate change (Lenoir et al. 2020; Parmesan and Yohe 2003; Rubenstein et al. 2023; Walther et al. 2002). However, a recent systematic review found that 37% of 6800 plant species globally are shifting downhill, or toward the equator (Osmolovsky et al. In review). This phenomenon is not unique to plants; 20%–24% of plant and animal taxa in terrestrial and marine habitats have exhibited counterintuitive shifts (Lenoir et al. 2010; Parmesan and Yohe 2003). While counterintuitive range shifts in species distributions have been predicted by species distribution models only once (Tagliari et al. 2021), the high proportion of species making counterintuitive shifts and the magnitude of these shifts suggest that they might reflect an important biological phenomenon. The aims of our study were: to explore the possibility that counterintuitive range shifts might result from changes in niche availability at the lower range edges, resulting from climate change induced changes in biotic interactions, as was proposed in the Interaction Opportunists Hypothesis (Osmolovsky et al. 2025); and to test how biotic interactions may change across the ranges of uphill‐shifting and non‐shifting species.

We focused on quantifying the release from herbivore damage as a driver of counterintuitive range shifts of plants. Many fundamental studies explore how herbivory changes across geographic and climatic gradients (Andrew and Hughes 2005; Andrew et al. 2012; Anstett et al. 2016; Hendrix and Marquis 1983; Kozlov et al. 2022; Moles et al. 2011; Pennings et al. 2009; Schemske et al. 2009). Although some studies suggest that herbivory may have little to no impact or even increase plants' fitness (Belsky 1986; Böttner et al. 2025; McArt et al. 2013), herbivory affects species survival, reproduction and dispersal capacities (Crawley 1989; Marquis 1984; Morris et al. 2007; Mothershead and Marquis 2000; Strauss et al. 1996), and contributes to range limits (Benning et al. 2019). Further, the change in herbivore pressure following introduction may play an important role in the process of enemy release, which is hypothesised to contribute to increased fitness, establishment, and further dispersal of many non‐native plants (Meijer et al. 2016; Xirocostas et al. 2023, 2025).

**FIGURE 1:**
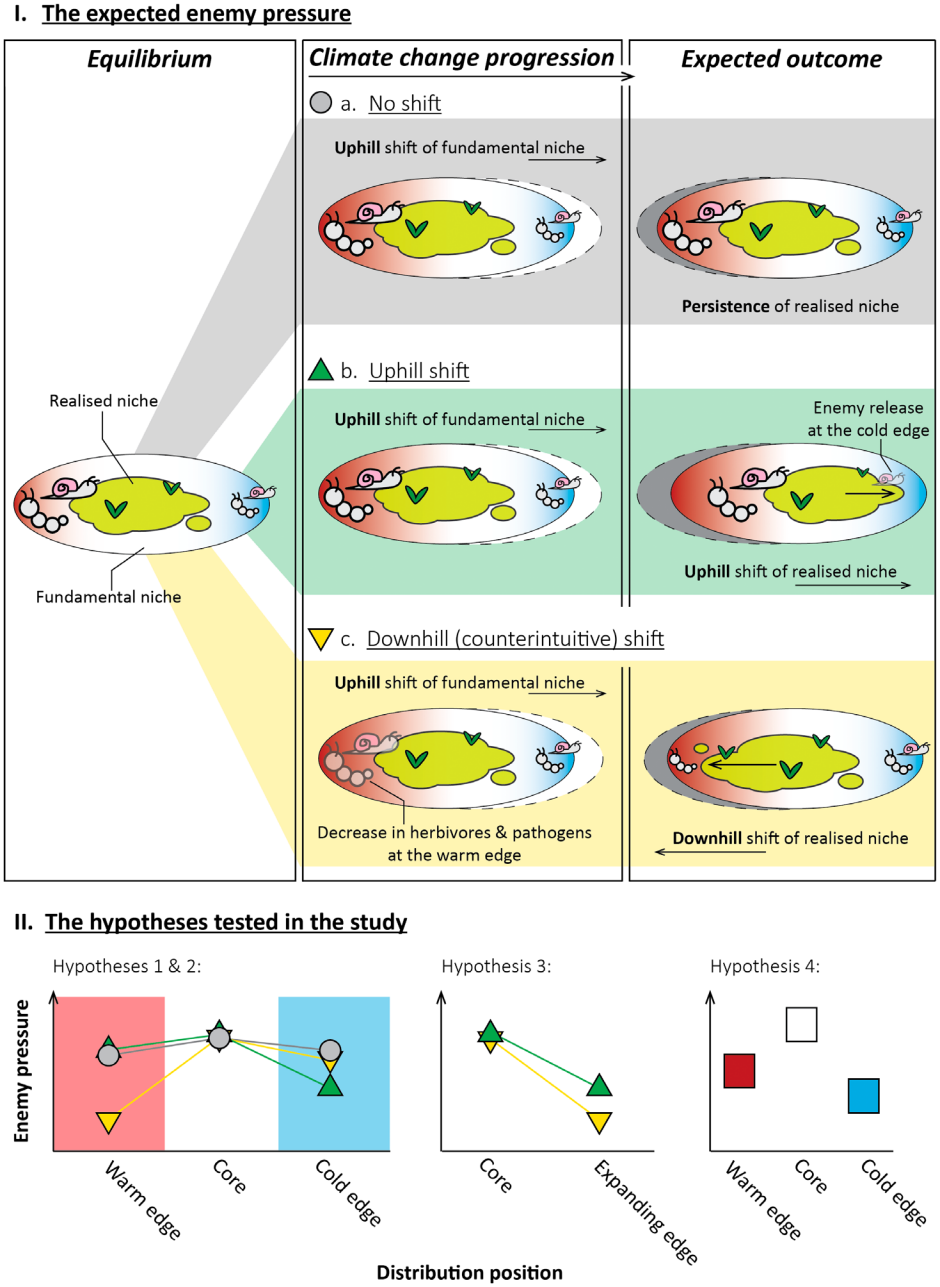
(I) The hypothesised enemy pressure (the amount and number of types of leaf damage) dynamics in species experiencing (a) no range shifts, (b) uphill shifts, or (c) downhill shifts in response to climate change, and (II) the graphic representation of the hypotheses tested in this study. In non‐shifting species (a), we expect no change in enemy pressure across the species ranges (Hypotheses 1, 2, and 4). In uphill‐shifting species (b), we expect lower enemy pressure due to release from enemies at the cold leading range edge (Hypotheses 1–3). We expect downhill‐shifting species (c) to experience lower enemy pressure at the warm range edge that will result in a downhill shift to previously unavailable parts of their fundamental niche (hypotheses 1–3).

We began by testing the hypothesis that downhill‐shifting plant species would experience lower amounts and fewer types of leaf damage at their warm (lower) range edge relative to the core of their distribution, compared to uphill‐shifting and non‐shifting species (Figure 1a and Figure 1: Hypothesis 1). Both theoretical (Dobzhansky 1950; Rasmann et al. 2014; Zvereva and Kozlov 2022) and empirical evidence (HilleRisLambers et al. 2013; Louthan et al. 2015; Paquette and Hargreaves 2021) suggest that antagonistic interactions may constrain the lower (warm) range edge of a species' distribution, despite suitable climatic conditions. The Interaction Opportunists Hypothesis (Osmolovsky et al. 2025) suggests that climate change may cause a decrease in antagonistic interactions (such as herbivory and pathogen attack) beyond a species' lower (warm) range edge. This decrease might allow the species to expand downhill and toward the equator, into parts of its fundamental niche that were previously unavailable, resulting in counterintuitive range shifts (Osmolovsky et al. 2025; Figure 1c). Such a scenario might arise if antagonists were more susceptible to climate change, or shifted their own ranges more quickly than the plant species (Lenoir et al. 2010; Osmolovsky et al. 2025; Tylianakis et al. 2008). Downhill range shifts in response to the climate change induced decrease in antagonistic interactions have been previously predicted (Foster and D'Amato 2015; Lawlor et al. 2024; Lenoir et al. 2010) but seldom tested. The most relevant evidence of which we are aware is reduced grazing leading to downhill range shifts in montane plants in the Himalayas (Bhatta et al. 2018), and reduced enemy pressure increasing introduced species' fitness, dispersal and establishment potential (Keane and Crawley 2002).

Next, we test the hypothesis that species shifting uphill experience lower amounts and fewer types of leaf damage at their cold range edge relative to their distribution core than do species that are shifting downhill or species that are not shifting (Figure 1b and Figure 1: Hypothesis 2). This prediction is based on the idea that species shifting upslope may experience enemy release when they colonise a new area (Keane and Crawley 2002). Some poleward and uphill‐shifting native species have shown decreased belowground and aboveground enemy pressure at their cold (leading) range edge (Cleavitt et al. 2022; Eskelinen et al. 2017; Kaarlejärvi et al. 2013; Morriën and van der Putten 2013; van Grunsven et al. 2010), and enemy release has been observed at the expanding edges of introduced species (Engelkes et al. 2008; Keane and Crawley 2002; Wan and Bonser 2016). In several cases, herbivory was also shown to be lower when plants were transplanted beyond the realised range on both elevational and latitudinal gradients (Andrew and Hughes 2007; Brown and Vellend 2014; Lakeman‐Fraser and Ewers 2013; Menéndez et al. 2008; Urli et al. 2016), although mammalian herbivory limited the establishment of lowland plants transplanted to high‐elevation habitats (Eskelinen et al. 2017; Kaarlejärvi et al. 2013).

We then posit that the difference in leaf damage between the expanding (warm) edge and the distribution core in downhill‐shifting species will be greater than the difference in herbivore pressure between the expanding (cold) edge and the distribution core of uphill‐shifting species. We predict this for both the amount of damage and the number of damage types (Figure 1: Hypothesis 3). We base our predictions on two underlying processes. First, as insects track climate change better than do plants (Bässler et al. 2013; Rubenstein et al. 2023), uphill‐shifting plants might experience only a moderate alleviation of biotic interaction strength (e.g., leaf damage), compared to their downhill and equatorward‐shifting counterparts. Second, while the current climate beyond the warm range edge may accommodate the species (Louthan et al. 2015; Lynn et al. 2021), the warm range edge is predicted to become less suitable with the onset of climate change, the release from enemy pressure needs to be strong enough to facilitate counterintuitive shifts (Chen et al. 2011; Loarie et al. 2009). Studies have already shown that positive biotic interactions could promote the persistence of plants in previously climatically unsuitable environments by dampening the effects of climate change (Anthelme et al. 2014; Ettinger and HilleRisLambers 2017). Similarly, we propose that a strong alleviation of enemy pressure could counteract the negative effects of climate on species' fitness. However, the release from enemies needs to be equal to or even stronger than the negative effect of climate change on the plants to promote downhill expansion.

Lastly, we test the hypothesis that both the total amount of damage and the number of damage types would decrease toward range edges, with higher leaf damage at the warm edge compared to the cold edge of the distribution (Figure 1a and Figure 1: Hypothesis 4). This prediction is based on two ideas. First, the strength of biotic interactions, and specifically herbivory, may become stronger at lower elevations and lower latitudes (Dobzhansky 1950; Hargreaves et al. 2014, 2019; Kozlov et al. 2022; Rasmann et al. 2014). However, evidence for this idea has been mixed (Anstett et al. 2016; Galmán et al. 2018; Moreira et al. 2018; Robinson et al. 2023). Second, herbivory may decrease toward all population edges (Alexander et al. 2007; García et al. 2000; Woods et al. 2012). That is, within a species' distribution, the edge effect is thought to be stronger than the latitudinal or elevational gradient (Anstett et al. 2016). Several studies that surveyed naturally occurring populations have found lower herbivory toward distribution edges than in core populations (Alexander et al. 2007; Crutsinger et al. 2013; García et al. 2000; Woods et al. 2012; but see: Andrew and Hughes 2005). Further, two transplant studies reported decreased herbivory beyond distribution edges in native tree species (Katz and Ibáñez 2016; Urli et al. 2016). However, other transplant studies show an increase in herbivory beyond population edges, suggesting that range edges may be constrained by antagonistic interactions (Andrew and Hughes 2007; Benning et al. 2019; Brown and Vellend 2014; Eskelinen et al. 2017; Kaarlejärvi et al. 2013; Lynn et al. 2021).

By exploring elevational range shifts in alpine plant species, we aim to improve understanding of how interactions between climate change and changes in biotic pressure might affect species' uphill and downhill (counterintuitive) range shifts in response to climate change. This insight can aid in inferring current and future species' responses to climate change, predicting future ecological communities' composition, and better mitigating the impacts of climate change on Earth's ecosystems.

## 2 Methods

### 2.1 Study Area

We sampled plants native to Kosciuszko National Park, southern New South Wales, Australia (36.43° S, 148.33° E). Study sites ranged from 1500 – 2210 m a.s.l. (see Appendix S1 for a full list of surveyed locations) encompassing sub‐alpine and alpine communities (Bear et al. 2006). The sub‐alpine vegetation is dominated by *Eucalyptus pauciflora* sub. *niphophila* woodlands, with areas of bog, fen, heath, and grasslands, while the vegetation in the alpine area is low‐growing shrubs, grasses, and forbs (Bear et al. 2006). The area has experienced a 0.02°C year^−1^ increase in annual temperature in the past 60 years (Auld et al. 2022; Hennessy et al. 2008; Sritharan et al. 2021).

### 2.2 Species Selection

We chose nine species from an empirical study on range shifts in 36 alpine plant species in Kosciusko National Park (Auld et al. 2022). We chose non‐deciduous species (accounting for long‐term herbivore damage) that displayed significant uphill and downhill shifts in mean elevation, and species that did not exhibit significant range shifts in any direction (Table 1). The distribution of five of the nine species spans Southeastern Australia (*Dichosciadium ranunculaceum* var. *ranunculaceum*, *Pappochroma setosum*, *Aciphylla glacialis*, *Nematolepis ovatifolia*, and *Prasophyllum tadgellianum*), one additionally grows in Tasmania (*Orites lancifolius*), two span mainland Australia, Tasmania, and New Zealand (*Pentachondra pumila* and *Lycopodium fastigiatum*), and one species is restricted to Kosciuszko National Park (*Ranunculus anemoneus*; Atlas of Living Australia 2026). The shifts in mean elevation of both uphill and downhill‐shifting species were partially driven by the expansion of their upper and lower range edges, respectively, as reported in Auld et al. (2022) and observed during our sampling.

**TABLE 1:** The list of the sampled species, their families, and the direction of their shift according to Auld et al. (2022), and our reanalysis.

Species	Family	Direction of shift
*Dichosciadium ranunculaceum* var. *ranunculaceum*	Apiaceae	Uphill 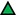
*Orites lancifolius*	Proteaceae	Uphill 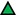
*Pappochroma setosum*	Asteraceae	Uphill 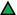
*Pentachondra pumila*	Ericaceae	Uphill 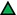
*Aciphylla glacialis*	Apiaceae	No shift 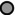
*Nematolepis ovatifolia*	Rutaceae	No shift 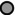
*Prasophyllum tadgellianum*	Orchidaceae	No shift 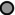
*Lycopodium fastigiatum*	Lycopodiaceae	Downhill 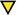
*Ranunculus anemoneus*	Ranunculaceae	Downhill 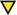

Only four species in Auld et al. (2022) showed significant downhill shifts, with one additional species exhibiting downhill contraction at its upper range edge. We were not able to locate the lowest reported distribution positions (1750, 1900 m) of one of the downhill‐shifting species (*Colobanthus affinis*), finding the lowest population 300 m above the lowest record (at 2090 m). Similarly, we were not able to locate the lowest reported position of *Carex pyrenaica* var. *cephalotes* (1384 m), as the record lacked a site description, and the coordinates were not close to an accessible road or track. Thus, we only sampled two of the five potential downhill‐shifting species (see Table 1). This is half of the downhill‐shifting species reported in Auld et al. (2022), providing a sufficient sample size to detect the role of herbivory in driving downhill range shifts.

We chose four uphill‐shifting species that were easily distinguishable from coexisting species, were flowering during the sampling season (late November–early March), and were abundant at the three target positions of their distribution we have sampled (especially the upper edge of their distribution for the uphill‐shifting species). For one of the species, *Orites lancifolius*, although we located the upper‐most recorded position (2065 m) again in 2024, it consisted of only one individual growing on the walking track. Thus, we sampled the next highest observed position (1940 m, Appendix S1).

We chose three non‐shifting species, opting for species that were easy to identify and whose distribution was resurveyed by Auld et al. (2022).

### 2.3 Measuring Leaf Damage

Sampling was carried out from November 2023 to February 2024. We sampled three distribution positions from each of the chosen species: the cold (upper) edge, the warm (lower) edge, and the core of their distribution (Appendix S1), ensuring that all positions of a species were measured within a maximum of seven days. We chose a core population as close as possible to the calculated centroid of the distribution. For *Aciphylla glacialis*, we sampled two distribution core positions (at 1810 and 1930 m, Appendix S1), as we could not identify a site closer to the distribution centroid. For each distribution position, we aimed to estimate leaf damage in 10 individuals; however, we sampled a position even when fewer individuals were present. At four positions, we could locate only 8–9 individuals (Table S1a). Further, in the case of the Vulnerable *Ranunculus anemoneus* (Department of the Environment 2026), we sampled one individual at the warm edge and four individuals at the distribution core (Table S1a). We ensured that the sampled individuals were located at least 5 m from any walking paths or roads, in all but one case. The lowest position of *Lycopodium fastigiatum* only grew on the edges of a footpath, disappearing completely at ~1 m in each direction from the footpath. At each site, we first randomly chose a direction, using a compass and a random number generator, sampling the first encountered individual in that direction. We repeated the process until 10 individuals were sampled, ensuring a distance of at least two canopy widths between one sampled individual to another (2–10 m, depending on the species). We never backtracked our steps to ensure every individual would be sampled only once. When individuals grew in mats or were hard to distinguish from other individuals, we sampled plants that grew 10 m apart as different individuals.

We aimed to assess leaf damage on 50 leaves across 10 branches per individual (see Table S1a for the number of leaves sampled). In shrubby species, we randomly chose 1–3 branches from four randomly selected directions; in species with leaves arranged in a rosette (with no distinct branches), we sampled 10 leaves from five randomly chosen directions, or all the available mature, but not senescent, leaves. In *Aciphylla glacialis* (which has compound leaves), we treated leaves as branches and considered leaflets as a leaf unit. On each branch, we aimed to estimate damage on five leaves, starting from the base of the branch. We skipped the first 1–2 viable leaves, stopping the estimation when five leaves were sampled or when leaves appeared juvenile (soft and/or with a different colour to the leaves at the base of the branch). In total, we collected observations on 11,294 leaves spanning nine species and 27 distribution positions, with 22–444 leaves sampled per position (Appendix S1).

For each leaf, we estimated both total leaf damage and the number of damage types (Appendix S2). We visually estimated seven damage types (mining, chewing, skeletal feeding, sap sucking, gall making, rasping, and fungal damage), following Andrew et al. (2012) and Castagneyrol et al. (2013). We ensured a match between the described damage types and how they appeared visually using the images from Labandeira et al. (2007). Leaf damage was visually estimated as the percent of damaged leaf, out of the total leaf area. We improved the accuracy and consistency of the measurements using the ZAX herbivory trainer (Xirocostas et al. 2022).

### 2.4 Statistical Analysis

All analyses were performed in R version 4.4.1 (R Core Team 2025), coupled with RStudio 2023.06 0.421 (Posit team 2025). We tested all the hypotheses by fitting a Generalised Linear Mixed Effect Model using the Template Model Builder package (*glmmTMB*; Brooks et al. 2017). We tested whether the warm and cold range edges differed from the distribution core in two separate models, allowing us to test the two hypotheses separately. To do that, we have subset the data into distribution core and warm edge, as well as distribution core and cold edge datasets.

To determine whether downhill‐shifting plant species experience less leaf damage (%) at their warm range edge relative to the distribution core than do uphill‐shifting and non‐shifting species (Hypothesis 1), we tested the relationship between the response variable—leaf damage (%), and the interaction between two explanatory variables—distribution position (warm edge or distribution core) and shift direction (uphill, downhill, or non‐shifting). We included a random intercept for sampled individuals nested within distribution position, nested within species. As the distribution of our data was right‐skewed with many zeros, we used a Tweedie distribution (Dunn et al. 2018), similar to a previous study comparing the extent of herbivore damage (Xirocostas et al. 2023):

$$\begin{array}{c} \text{Leaf damage}\;\left(\%\right)\sim {\text{position}}_{\left(\text{warm edge}/\text{core}\right)}\\ \;\times {\text{shift direction}}_{\left(\text{uphill}/\text{downhill}/\mathrm{no}\right)}\\ \;+\left(1|\text{species}/\text{position}/\text{individual}\right),\text{family}=\text{Tweedie} \end{array}$$

Next, we tested the difference in the number of damage types experienced at the warm range edge versus the distribution core was lower in downhill‐shifting species than in uphill‐shifting and non‐shifting species (Hypothesis 1) using a similar model, with the number of damage types as the response variable. As the number of damage types are counts, we fitted a Poisson distribution (Zuur et al. 2009):

$$\begin{array}{c} \text{Number of damage types}\sim {\text{position}}_{\left(\text{warm edge}/\text{core}\right)}\\ \;\times {\text{shift direction}}_{\left(\text{uphill}/\text{downhill}/\mathrm{no}\right)}\\ \;+\left(1|\text{species}/\text{position}/\text{individual}\right),\text{family}=\text{Poisson} \end{array}$$

We used similar models to determine whether the leaf damage and the number of damage types experienced at the upper cold range edge versus the distribution core was lower in uphill‐shifting species compared to downhill‐shifting and non‐shifting species (Hypothesis 2):

$$\begin{array}{c} \text{Leaf damage}\;\left(\%\right)\sim {\text{position}}_{\left(\text{cold edge}/\text{core}\right)}\\ \;\times {\text{shift direction}}_{\left(\text{uphill}/\text{downhill}/\mathrm{no}\right)}\\ \;+\left(1|\text{species}/\text{position}/\text{individual}\right),\text{family}=\text{Tweedie} \end{array}$$

$$\begin{array}{c} \text{Number of damage types}\sim {\text{position}}_{\left(\text{cold edge}/\text{core}\right)}\\ \;\times {\text{shift direction}}_{\left(\text{uphill}/\text{downhill}/\mathrm{no}\right)}\\ \;+\left(1|\text{species}/\text{position}/\text{individual}\right),\text{family}=\text{Poisson} \end{array}$$

Next, we tested if leaf damage and the number of damage types experienced at the expanding range edge relative to the distribution core were lower for downhill‐shifting species than for uphill‐shifting species (Hypothesis 3). We included an interaction between distribution position (expanding/distribution core) and shift direction (uphill/downhill) as the explanatory variables. Additionally, we included a random intercept for individuals nested within distribution position, nested within species:

$$\begin{array}{c} \text{Leaf damage}\;\left(\%\right)\sim {\text{position}}_{\left(\text{expanding edge}/\text{core}\right)}\\ \;\times {\text{shift direction}}_{\left(\text{uphill}/\text{downhill}\right)}\\ \;+\left(1\;\text{species}/\text{position}/\text{individual}\right),\text{family}=\text{Tweedie} \end{array}$$

$$\begin{array}{c} \text{Number of damage types}\sim {\text{position}}_{\left(\text{expanding edge}/\text{core}\right)}\\ \;\times {\text{shift direction}}_{\left(\text{uphill}/\text{downhill}\right)}\\ \;+\left(1|\text{species}/\text{position}/\text{individual}\right),\text{family}=\text{Poisson} \end{array}$$

In all models, we were interested in the interaction term between position and direction of shift (see Appendix S3 for full model results). We used type III ANOVA from the *car* package to calculate the significance level of the interaction term (Fox and Weisberg 2019), and the *emmeans* package to perform post hoc tests and calculate effect sizes (Lenth 2023).

Lastly, we tested whether there was less leaf damage and fewer damage types at the cold and warm edges than in the distribution core (Hypothesis 4). To avoid confounding our results with changes in damage due to range shifts, we only test this hypothesis on species that exhibited no significant range shifts in response to climate change. We used leaf damage and the number of damage types as response variables in two separate models, with position (cold edge, warm edge, or distribution core) as the explanatory variable in both models. We also included a random intercept for sampled individuals nested within species in both models.

$$\begin{array}{c} \text{Leaf damage}\;\left(\%\right)\sim {\text{position}}_{\left(\text{cold edge}/\text{warm edge}/\text{core}\right)}\\ \;+\left(1|\text{species}/\text{individual}\right),\text{family}=\text{Tweedie} \end{array}$$

$$\begin{array}{c} \text{Number of damage types}\sim {\text{position}}_{\left(\text{cold edge}/\text{warm edge}/\text{core}\right)}\\ \;+\left(1|\text{species}/\text{individual}\right),\text{family}=\text{Poisson} \end{array}$$

For all models, we calculated marginal and conditional *R* ^2^ values using the *easystats* package (Lüdecke et al. 2022; Nakagawa and Schielzeth 2013). The damage type data were not overdispersed (tested using the *check_overdispersion* function from the *performance* package (Lüdecke et al. 2021); *p* = 1 for all models). Further, the assumptions for the linear distribution of residuals and the homogeneity of the variability were tested using the *DHARMa* package (Hartig 2022). We used the *ggplot2* package for all data visualisation (Wickham 2016). For hypotheses 1–3, we displayed the estimated mean and confidence intervals of the amount and number of types of herbivore damage, for each distribution position of uphill, non‐shifting, and downhill‐shifting plants, using the *emmeans* package (Lenth 2023). We additionally tested if there is a significant difference in the damage percentage and number of damage types between different populations within each species, using the *glmmTMB*, *emmeans*, and *car* packages (Brooks et al. 2017; Fox and Weisberg 2019; Lenth 2023). We present leaf damage (%) and the number of damage types at the three distribution positions for each of the species and the post hoc results of the statistical analyses (see Appendices S3 and S4 for the full results). To present the data, we log‐transformed the percentage of leaf damage, adding half of the smallest value to represent zeros on the log‐scale (Warton and Hui 2011).

## 3 Results

**FIGURE 2:**
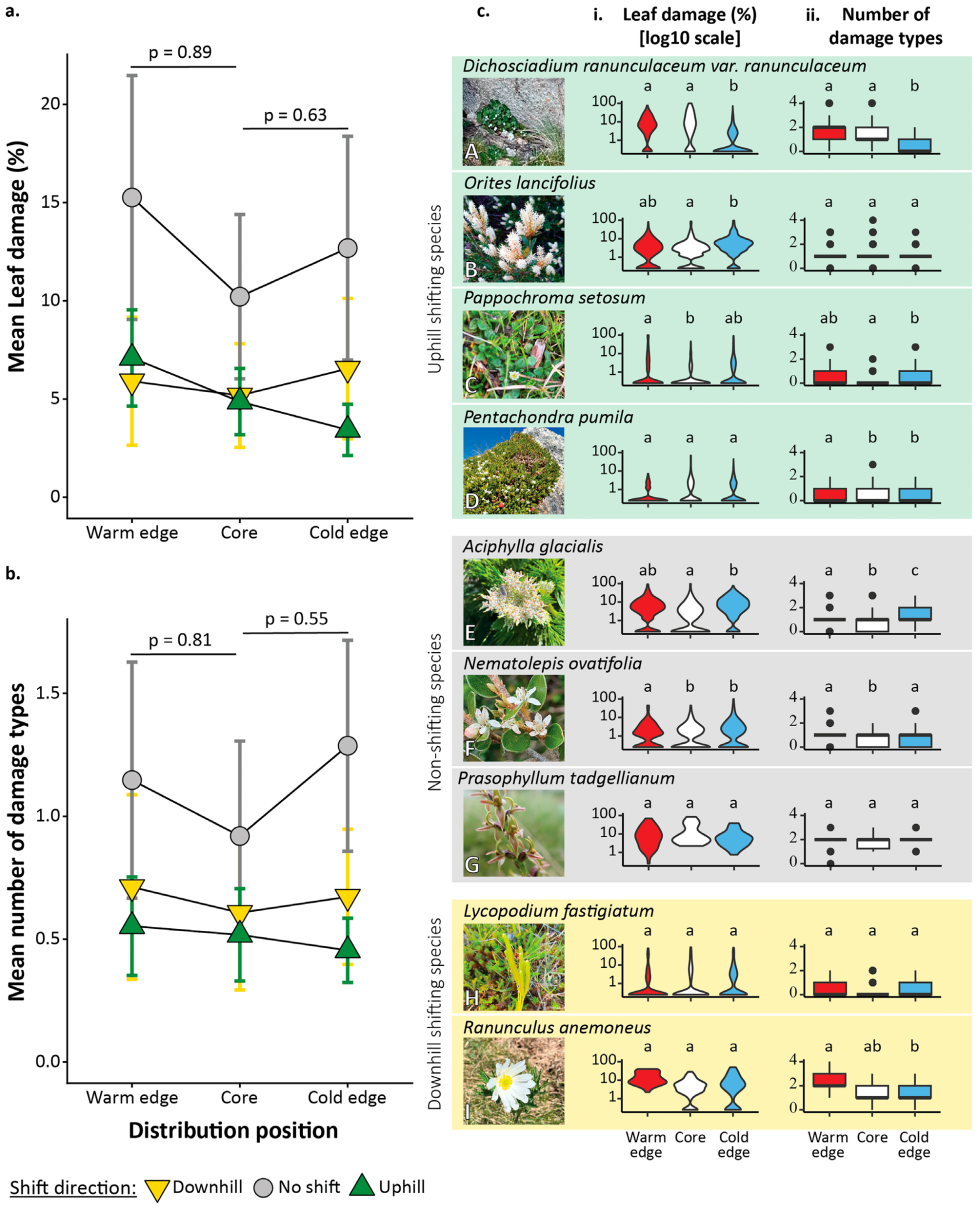
The mean percentage of leaf damage (a), and the mean number of damage types (b) at the three distribution positions of downhill (yellow downturned triangle), uphill (green upturned triangle), and non‐shifting (grey circle) plant species. The triangles and circles represent leaf damage (a), or the number of damage types (b) at each distribution position, per shift direction. The error bars span ± standard error. *p*‐values above the figures denote the statistical significance of the interaction between shift direction and distribution position. Panel (c) presents the percentage of leaf damage (i) and the number of types of damage (ii) for each species at each of the distribution positions—warm edge (red), distribution core (white), and cold edge (blue). The violin plots denote the distribution of the percentage of leaf damage (i). The boxplots span the 25th to 75th quantiles of the number of types of damage (ii), with the whiskers spanning the 5th to the 95th quantiles, and the central line denoting the median. Letters above the violin plots and boxplots denote the statistical significance of pairwise comparisons of leaf damage (%) and the number of damage types between distribution positions for each species (see Appendices S3 and S4 for the full results, and Appendix S5 for the average leaf damage values in each distribution position and across elevation). Photos A, C, E, & J were taken by I. Osmolovsky, photo D was taken by A. T. Moles, photos B, F, and H were taken by V. Williamson, and photo G by M. Mallen‐Cooper.

Contrary to our first hypothesis, we found no evidence of a difference in leaf damage (%) at the warm range edge relative to the distribution core in downhill‐shifting compared to uphill‐shifting and non‐shifting species (*p* = 0.89, *R* ^2^ = 0.07; Figure 2a). Similarly, there was no evidence for a difference in the relative number of damage types at the warm edges versus distribution core between uphill, downhill, and non‐shifting species (*p* = 0.81, *R* ^2^ = 0.06; Figure 2b).

Contrary to our second hypothesis, leaf damage (%) and the number of damage types experienced at the cold (upper) range edge versus the distribution core did not differ in uphill, in comparison to downhill and non‐shifting species (leaf damage (%): *p* = 0.63, *R* ^2^ = 0.10; Number of damage types: *p* = 0.55, *R* ^2^ = 0.10; Figure 2a–c).

**FIGURE 3:**
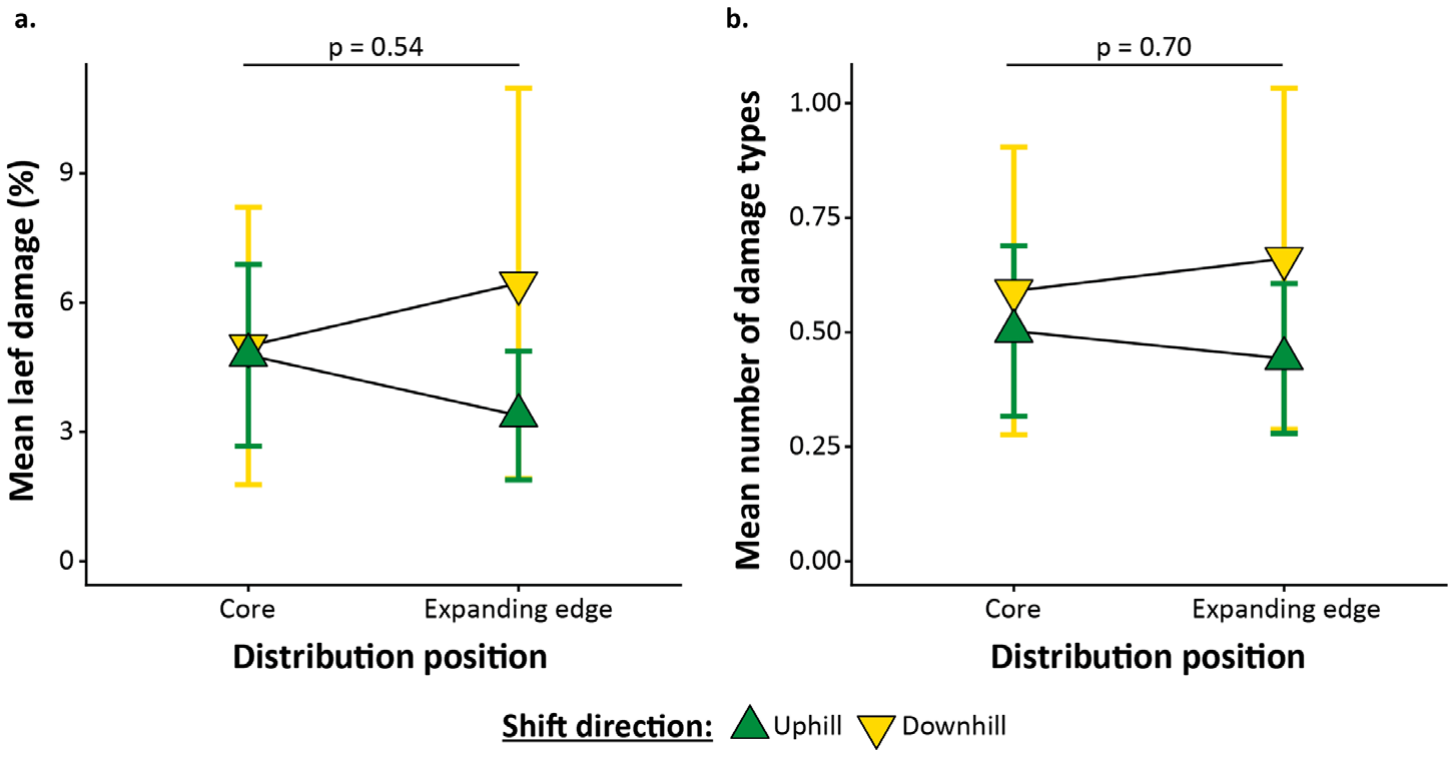
The mean percentage of leaf damage (a) and the mean number of damage types (b) at the distribution core and expanding edges of downhill (green downturned triangle) and uphill (yellow upturned triangle) shifting species. The triangles represent the mean leaf damage percentage (a) and number of damage types (b), with the error bars representing ± standard error. The *p*‐values above the figures denote the statistical significance of the interaction between position and direction of shift.

Downhill‐shifting plants did not experience lower leaf damage or a lower number of damage types at the expanding range edge versus distribution core than uphill‐shifting plants, contradicting our third hypothesis (leaf damage (%): *p* = 0.54, *R* ^2^ = 0.02, Figure 3a; number of damage types: *p* = 0.70, *R* ^2^ = 0.01; Figure 3b).

**FIGURE 4:**
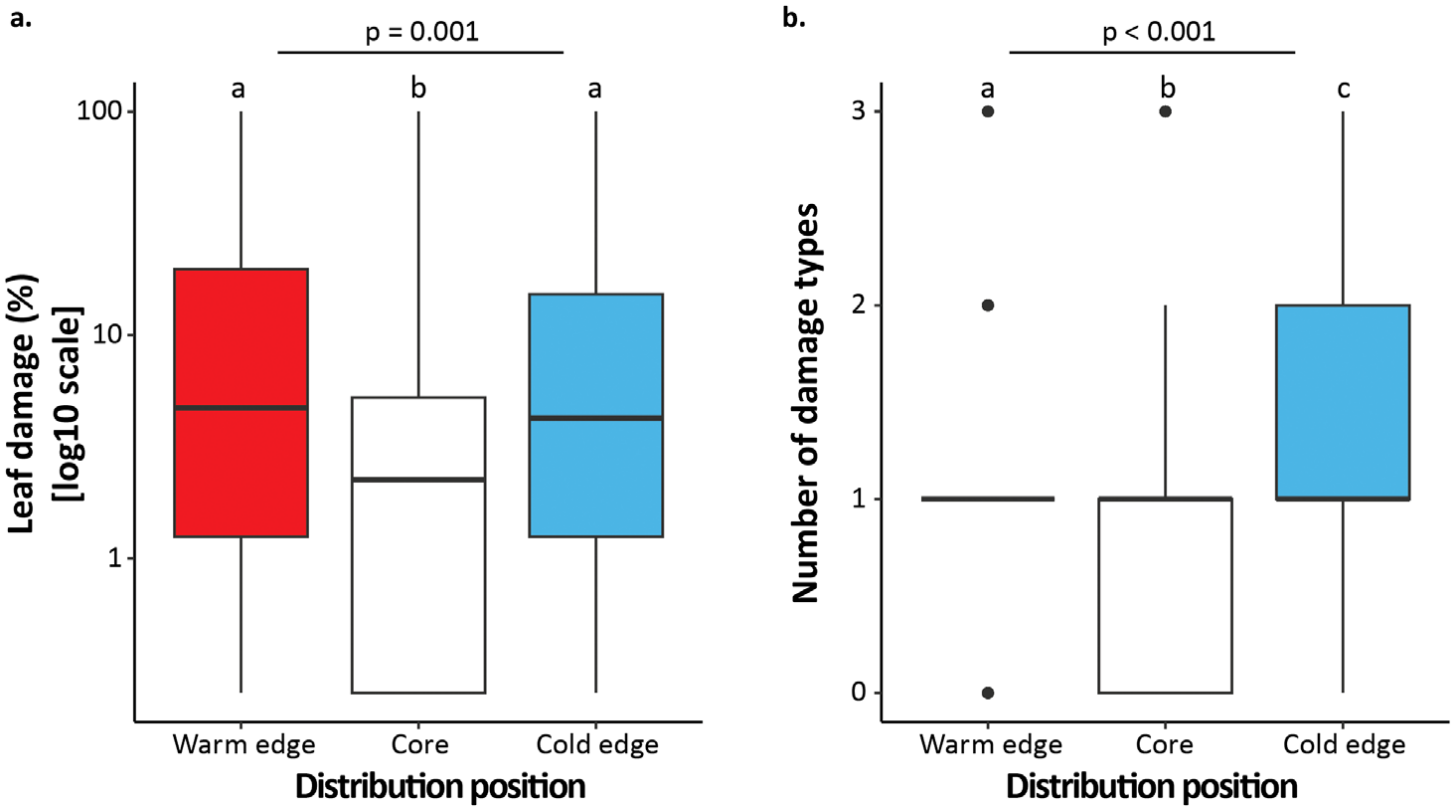
The percentage of leaf damage (a) and number of damage types (b) at the lower (red) and upper (blue) edges and the distribution core of the distribution (white) of non‐shifting species. The boxes within each boxplot span the 25th to the 75th quantiles, whiskers span the 5th to the 95th quantiles, and the central line represents the median. The *p*‐values above the figures denote the statistical significance of the interaction between position and direction of shift. The letters above the boxplots denote the statistical significance of the pairwise comparison of the leaf damage (%) and the number of damage types between distribution positions.

Finally, there was a difference in both the amount of leaf damage (*p* = 0.001, *R* ^2^ = 0.03) and the number of damage types (*p* < 0.001, *R* ^2^ = 0.05) experienced by non‐shifting plants at different distribution positions. However, the trend was opposite to our fourth hypothesis for both leaf damage and the number of damage types. Species experienced 6.4% and 4.5% more leaf damage at the warm and cold range edges than at the core (*p*_Tukey_ = 0.001 and 0.02, respectively; Figure 4a). The number of damage types differed substantially between all distribution positions (*p*_Tukey_ ≤ 0.05), with plants at distribution core experiencing a mean of 0.9 damage types while plants at the warm and cold edges experienced a mean of 1.2 and 1.4 damage types (Figure 4b).

## 4 Discussion

Our data do not support the Interaction Opportunists Hypothesis (Osmolovsky et al. 2025), which proposes that counterintuitive range shifts may be driven by a climate change‐induced decrease in antagonistic interactions at the warm range edge. Downhill‐shifting species in our study do not experience relatively less leaf damage at their warm range edge than at their range core than do non‐shifting or uphill‐shifting species. We can think of at least five explanations for these unexpected results: (1) The Interaction Opportunists Hypothesis might not apply as widely as originally hypothesised; (2) Counterintuitive shifts are driven by biotic interactions other than leaf damage; (3) Although plants did not experience less leaf damage at the warm range edge than at their distribution cores, a change in enemy pressure through time might still have occurred, explaining the downhill range shifts; (4) The counterintuitive shift of some of the downhill plants in our study may be driven by anthropogenic activity/disturbance, with human activity contributing to the biotic suitability of the habitat; (5) herbivory may be contributing to counterintuitive range shifts, but this pattern is not detected because of the small effective sample size in our study. We address each of these possibilities below.

The Interaction Opportunists Hypothesis (Osmolovsky et al. 2025) is based in part on the premise that biotic interactions are stronger and may constrain species at warm range edges. While there is some evidence for latitudinal and elevational gradients in biotic interactions (Dobzhansky 1950; Hargreaves et al. 2019; HilleRisLambers et al. 2013; Louthan et al. 2015; Paquette and Hargreaves 2021; Zvereva and Kozlov 2022) many empirical studies and data compilation studies find no significant elevational or latitudinal gradients in herbivory, or higher herbivory at higher elevations or latitudes (Galmán et al. 2018; Moles et al. 2011). Similarly, we did not find a difference in leaf damage or the number of damage types experienced at high versus low elevations (Figure 4 and Appendix S5). While biotic interactions may constrain the warm edge of species' distributions (HilleRisLambers et al. 2013; Paquette and Hargreaves 2021), biotic interactions can also constrain the cold range edge. American beech ( *Fagus grandifolia* ) was able to shift uphill faster where enemy abundance was reduced at its upper (cold) range edge, suggesting that the presence of herbivores might have previously limited this species' upper range edge (Cleavitt et al. 2022). Pollination can also limit plant species' upper range edges (HilleRisLambers et al. 2013; Moeller et al. 2012). On the other hand, climate rather than biotic interactions may limit species distributions at the warm range edge. For example, changes in local precipitation drove downhill shifts in plants across mountain ranges in California, South Africa, and China (Crimmins et al. 2011; Fu et al. 2024; Zu et al. 2022). Climate constraining species' distributions at the warm range edge, and the lack of strong evidence for an elevational gradient in herbivore and leaf pathogen interactions, together with our findings, suggest that factors other than antagonistic biotic interactions may drive counterintuitive range shifts. For example, antagonistic and mutualistic biotic interactions other than herbivory and leaf pathogens might drive counterintuitive shifts by creating suitable conditions downhill of the current distribution or reducing forces limiting lower distribution. Potentially important interactions include decreases in intra‐ and interspecific competition, predation, and parasitism, and/or increases in above‐ and/or belowground positive interactions (Lenoir et al. 2010; Osmolovsky et al. 2025). Thus, we believe it would be overly hasty to throw the Interactions Opportunists hypothesis out based on a study of one interaction type in one ecosystem. We advocate for more studies on a range of types of biotic interactions in a range of different ecosystems.

Lower herbivory at the warm edge versus core of downhill shifting species, compared to uphill and non‐shifting plants may not result in lower leaf damage, as we predicted. If before the onset of climate change, plants experienced higher leaf damage at their lower (warm) range edges than in other parts of their distribution (Anstett et al. 2016; Hargreaves et al. 2014; HilleRisLambers et al. 2013; Paquette and Hargreaves 2021), leaf damage may have prevented plants from expanding into climatically suitable niches downhill. The effects of climate change could then contribute to a lower herbivory, to a level at which leaf damage no longer precludes species' establishment beyond their realised niche, allowing downhill range expansions to occupy a greater proportion of their fundamental niche. The resulting levels of leaf damage may appear uniform, or similar between the newly expanded warm edge and distribution core, despite a decrease through time in enemy pressure that has occurred. It would be worthwhile testing this idea in future studies by resampling previously measured climatic gradients, latitudinal gradients and elevational gradients in leaf damage to see whether there have been consistent changes in damage across species ranges through time.

Our results may indicate that some downhill range shifts are driven by human disturbance and/or by changes in disturbance regimes. Disturbance has been proposed as a potential explanation for counterintuitive shifts in other montane ecosystems (Foster and D'Amato 2015; Lenoir et al. 2010). Further, human disturbance can affect biotic interactions (e.g., reducing competitor pressure), prompting counterintuitive shifts indirectly (Bhatta et al. 2018; Lenoir et al. 2010). One of the species in our study, *L. fastigiatum*, might be undergoing a downhill shift as a result of human activity. We observed that at the lowest part of its distribution, *L. fastigiatum* only grows within ~50 cm of a footpath. The habitat in that area is characterised by relatively tall (> 1.5 m), and dense vegetation, whereas at all other parts of its distribution, we observed *L. fastigiatum* growing in habitats with mostly short vegetation (10–15 cm), regardless of proximity or presence of footpaths. Thus, the walking trail might provide a suitable habitat for *L. fastigiatum*, by reducing light competition with the high vegetation abundant in the area. Other studies have shown that proximity to roads might promote faster range shifts of both native and introduced species (Dainese et al. 2017; Lembrechts et al. 2017), potentially contributing to their survival in the face of climate change. However, we did not observe similar circumstances that could drive a downhill shift of *R. anemoneous*, and thus, factors other than human activity may drive this species to shift downhill. Many conservation efforts are focused on conserving ‘pristine’ and ‘intact’ environments (Plumptre et al. 2021; Soulé 1985). However, we propose that conserving pristine environments is not just unattainable but might contribute to the extirpation or extinction of some species. Our observation of the habitat at the low range edge of *L. fastigiatum* suggests that moderate levels of human activity might be crucial to the survival of certain species.

Our results suggest that the Interaction Opportunists Hypothesis does not always explain counterintuitive range shifts. Of course, it is not unusual for ecological hypotheses not to apply in all cases (Mackey and Currie 2001; Maestre et al. 2009). Thus, we do not feel that it is appropriate to abandon the hypothesis after testing in a single system. If repeated tests fail to support the Interactions Opportunists hypothesis, it will need to be abandoned. What seems more likely is that repeated tests will shine light on the circumstances under which the Interaction Opportunists hypothesis applies. In this latter case, the hypothesis should be re‐examined and revised. Similarly, other fundamental ecological hypotheses are often revised and re‐examined after multiple studies return contrasting results. For example, the Stress Gradient Hypothesis, which suggests that antagonistic interactions may become positive under increased environmental stress, was at first thought to apply globally (Bertness and Callaway 1994). However, following mixed support for the hypothesis from multiple studies and systematic data compilations (Adams et al. 2022; Lortie and Callaway 2006; Maestre et al. 2009, 2005), it has been revised more recently to apply in a narrower set of conditions, for example, in less stressful environments (Holmgren and Scheffer 2010). Other ecological theories, such as the existence of a latitudinal gradient in biotic interactions (Dobzhansky 1950; Schemske et al. 2009) and the Intermediate‐Disturbance Hypothesis (Connell 1978), have similarly undergone revisions and re‐examinations (Anstett et al. 2016; Fox 2013; Mackey and Currie 2001; Moles et al. 2011).

Contrary to our hypothesis, non‐shifting species exhibited higher leaf damage and more damage types at the range edges than in the core (Figure 4). While our results are not in line with the theory that herbivore pressure would be lower toward distribution edges (Anstett et al. 2016), several transplant studies report increased herbivore and granivore pressure at and beyond range edges (Benning et al. 2019; Brown and Vellend 2014; Lynn et al. 2021). We propose that the observed damage pattern may be driven by positive density dependence, similar to that observed in predator satiation during masting events (Kelly and Sork 2002; Pesendorfer et al. 2021). If species are highly abundant in the distribution core, they may outnumber the available herbivores, leading to lower herbivory per individual (Janzen 1971; Solomon 1949). In contrast, the fewer individuals at range edges may experience overall higher herbivory rates. Another possibility is that there may be an elevational change in the types and abundance of predators. For example, small‐seeded plant species in the Andes experience high seed predation by ants at the lower range edge, and high predation by granivorous mammals at the upper edge of their distribution (Hillyer and Silman 2010). Both ants and mammals were less abundant at mid‐elevations, contributing to low seed predation at the distribution core (Hillyer and Silman 2010). The species in our study may similarly experience an elevational change in the types and abundance of enemies they encounter.

A final possibility is that the sample size in our study was too small to detect a substantial change in herbivory across downhill‐shifting species ranges. While we have observed herbivore damage on 11,294 leaves, our sampling was only spread across three distribution positions of two downhill, three non‐shifting, and three uphill‐shifting species. Although we aimed to sample as many individuals as possible in each position, in many of them, the number of individuals was ten or lower. Thus, it may be possible that sampling more species and more individuals within each distribution position could provide different results. Further, sampling multiple species could pinpoint whether biotic interactions are the strongest driver of counterintuitive range shifts. Nonetheless, previous studies that have explored changes in biotic interaction across geographic and climatic gradients have focused on one species at a time, often reporting differing and contradicting patterns (Ettinger et al. 2011; Ettinger and HilleRisLambers 2013; Hargreaves et al. 2015; HilleRisLambers et al. 2013). Further, our study is the first, to our knowledge, to test the Interaction Opportunists hypothesis, and further studies may provide more evidence, confirming or contradicting the proposed hypothesis.

Our results were not consistent with the idea that leaf damage underpins counterintuitive range shifts in plants. The question of what could drive the counterintuitive range shifts commonly observed worldwide remains. Despite the recent increase in studies exploring counterintuitive range shifts (Crimmins et al. 2011; Fu et al. 2024; Kuhn et al. 2016; Neate‐Clegg et al. 2021; Pinsky et al. 2013; Zu et al. 2022), our understanding of potential drivers is limited to climatic variables, topography, and plant traits. We need additional empirical information as well as theory on factors that might affect how species respond to climate change. Such information would improve the accuracy of the predictions of species distribution models (Booth 2018) and increase the potential for positive conservation outcomes.

## Author Contributions

**Inna Osmolovsky:** conceptualization (lead), formal analysis (lead), funding acquisition (equal), investigation (lead), methodology (lead), project administration (lead), visualization (lead), writing – original draft (lead), writing – review and editing (equal). **Zoe A. Xirocostas:** investigation (equal), methodology (equal), writing – review and editing (equal). **Giancarlo M. Chiarenza:** investigation (equal), methodology (equal), writing – review and editing (equal). **Eve Slavich:** formal analysis (equal), visualization (equal), writing – review and editing (equal). **Angela T. Moles:** conceptualization (supporting), formal analysis (supporting), funding acquisition (equal), methodology (supporting), supervision (lead), visualization (supporting), writing – original draft (supporting), writing – review and editing (equal).

## Funding

This work was supported by the Ecological Society of Australia, Student Research Grant and Australian Research Council (DP180103611).

## Conflicts of Interest

The authors declare no conflicts of interest.

## Supporting information


**Appendix S1:** A list of all sampled locations per species, their coordinates and altitude, and the number of sampled individuals.
**Appendix S2:** The types of damage recorded during the survey.
**Appendix S3:** Full statistical model results.
**Appendix S4:** Statistical analysis and pairwise comparison of leaf damage (%) and the number of damage types between positions, for individual species.
**Appendix S5:** Summary of the mean leaf damage values across the distribution position of the sampled species.

## Data Availability

Code and data associated with this study are publically available on Figshare through the following link: https://doi.org/10.6084/m9.figshare.28561064.
