# Supplementary material for: Leaf Damage is Not the Answer! Reduced Herbivore Pressure Does Not Underpin Either Downhill or Uphill Range Shifts
Source: Ecol Evol. 2026 Apr 6;16(4):e73437. doi: 10.1002/ece3.73437 (PMC13051979; doi:10.1002/ece3.73437)

Supplementary material

**Appendix S1.** A list of all sampled locations per species, their coordinates and altitude, and the number of sampled individuals

**Appendix S2.** The types of damage recorded during the survey

**Appendix S3.** Full statistical model results

**Appendix S4.** Statistical analysis and pairwise comparison of leaf damage (%) and the number of damage types between positions, for individual species

**Appendix S5.** Summary of the mean leaf damage values across the distribution position of the sampled species

**Appendix S1.** The list of sampled locations for each species

*Table S1a. The list of sampled locations for each species, the sampling date, the elevation and coordinates, and the number of individuals sampled in each position.*

| **Species** | **Sampling date** | **Position** | **Elevation** | **Latitude** | **Longitude** | **N individuals sampled** | **N leaves sampled** |
| --- | --- | --- | --- | --- | --- | --- | --- |
| *Aciphylla glacialis* | 3/12/2023 | Warm | 1760 | -36.4283 | 148.319722 | 10 | 411 |
| *Aciphylla glacialis* | 30/11/2023 | Core | 1810 | -36.4319 | 148.329722 | 10 | 311 |
| *Aciphylla glacialis* | 4/12/2023 | Core | 1930 | -36.4161 | 148.311111 | 8 | 266 |
| *Aciphylla glacialis* | 5/12/2023 | Cold | 2210 | -36.4572 | 148.263333 | 10 | 444 |
| *Dichosciadium ranunculaceum* var. *ranunculaceum* | 30/12/2023 | Warm | 1860 | -36.4089 | 148.3197222 | 10 | 395 |
| *Dichosciadium ranunculaceum* var*. ranunculaceum* | 3/01/2024 | Core | 1940 | -36.4406 | 148.3252778 | 10 | 325 |
| *Dichosciadium ranunculaceum* var. *ranunculaceum* | 23-31/12/2023 | Cold | 2150 | -36.4561 | 148.2669444 | 10 | 169 |
| *Lycopodium fastigiatum* | 4/01/2024 | Warm | 1470 | -36.5164 | 148.2816667 | 10 | 132 |
| *Lycopodium fastigiatum* | 3/01/2024 | Core | 1950 | -36.4403 | 148.3247222 | 10 | 120 |
| *Lycopodium fastigiatum* | 12/01/2024 | Cold | 2200 | -36.4569 | 148.2630556 | 9 | 179 |
| *Nematolepis ovatifolia* | 1/12/2023 | Warm | 1730 | -36.4308 | 148.322222 | 10 | 404 |
| *Nematolepis ovatifolia* | 1/12/2023 | Core | 1830 | -36.4317 | 148.327778 | 10 | 336 |
| *Nematolepis ovatifolia* | 6/12/2023 | Cold | 2060 | -36.4506 | 148.279444 | 10 | 372 |
| *Orites lancifolius* | 24/12/2023 | Warm | 1390 | -36.5092 | 148.2966667 | 10 | 381 |
| *Orites lancifolius* | 24/12/2023 | Core | 1900 | -36.4453 | 148.3127778 | 10 | 413 |
| *Orites lancifolius* | 12/01/2024 | Cold | 1940 | -36.4372 | 148.3247222 | 8 | 365 |
| *Pappochroma setosum* | 30/12/2023 | Warm | 1870 | -36.4117 | 148.3211111 | 10 | 108 |
| *Pappochroma setosum* | 27/12/2023 | Core | 1960 | -36.4422 | 148.3252778 | 10 | 61 |
| *Pappochroma setosum* | 1/01/2024 | Cold | 2090 | -36.4792 | 148.2733333 | 10 | 162 |
| *Pentachondra pumila* | 29/01/2024 | Warm | 1920 | -36.43 | 148.3205556 | 10 | 143 |
| *Pentachondra pumila* | 1/02/2024 | Core | 2030 | -36.4347 | 148.3227778 | 10 | 215 |
| *Pentachondra pumila* | 31/01/2024 | Cold | 2210 | -36.4572 | 148.263333 | 10 | 196 |
| *Prasophyllum tadgellianum* | 28/12/2023 | Warm | 1720 | -36.4303 | 148.3213889 | 10 | 37 |
| *Prasophyllum tadgellianum* | 27/12/2023 | Core | 1950 | -36.44 | 148.3247222 | 10 | 22 |
| *Prasophyllum tadgellianum* | 7/01/2024 | Cold | 2190 | -36.4544 | 148.2627778 | 10 | 29 |
| *Ranunculus anemoneus* | 10/01/2024 | Warm | 1740 | -36.4283 | 148.3197222 | 1 | 33 |
| *Ranunculus anemoneus* | 10/01/2024 | Core | 1870 | -36.4336 | 148.3955833 | 4 | 78 |
| *Ranunculus anemoneus* | 11/01/2024 | Cold | 2120 | -36.4486 | 148.2669444 | 9 | 61 |

**Appendix S2.** A table of the types of damage recorded during the survey

*Table S2a. The types of herbivory damage recorded during the survey, and the photos used to identify each type. Photo credit: I. Osmolovsky and Z.A. Xirocostas*

| **Herbivory type** | **Photo** |
| --- | --- |
| Mining | 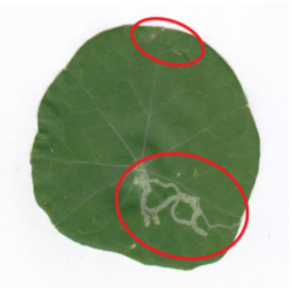 |
| Chewing | 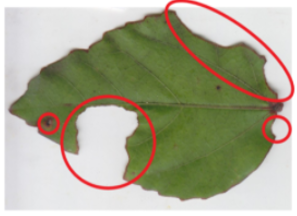 |
| Skeletal feeding | 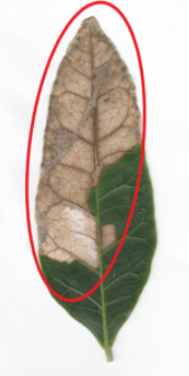 |
| Sap sucking | 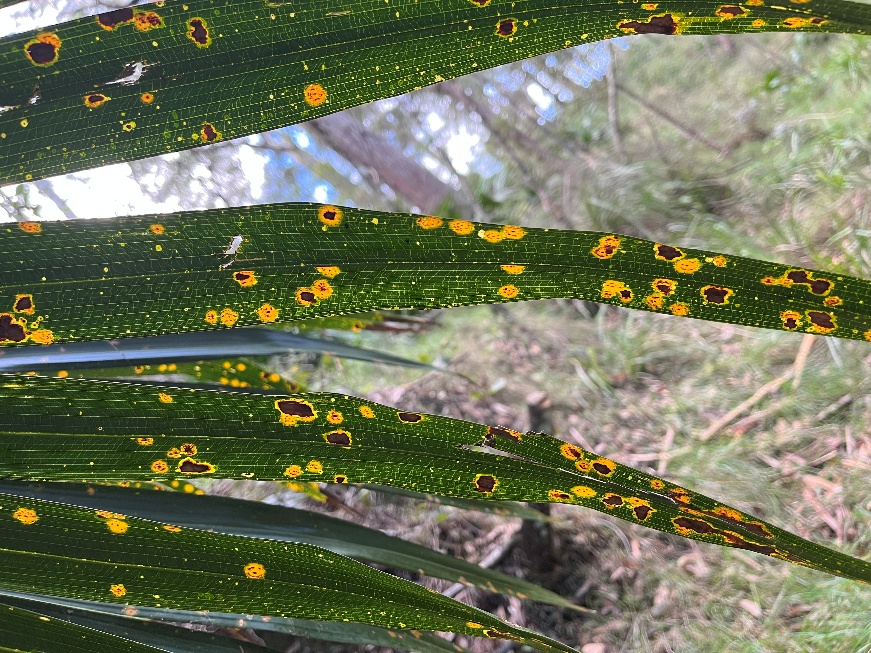 |
| Gall making | 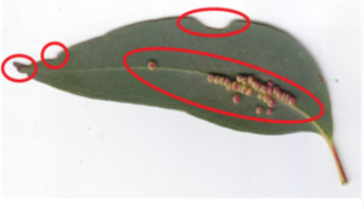 |
| Rasping | 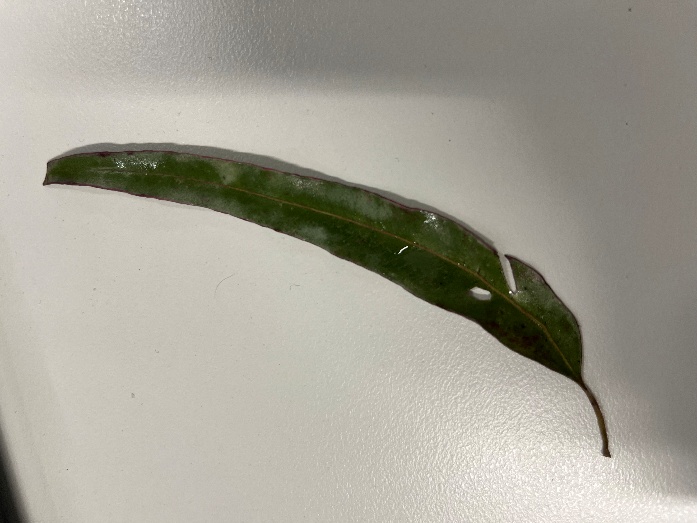 |
| Fungi | 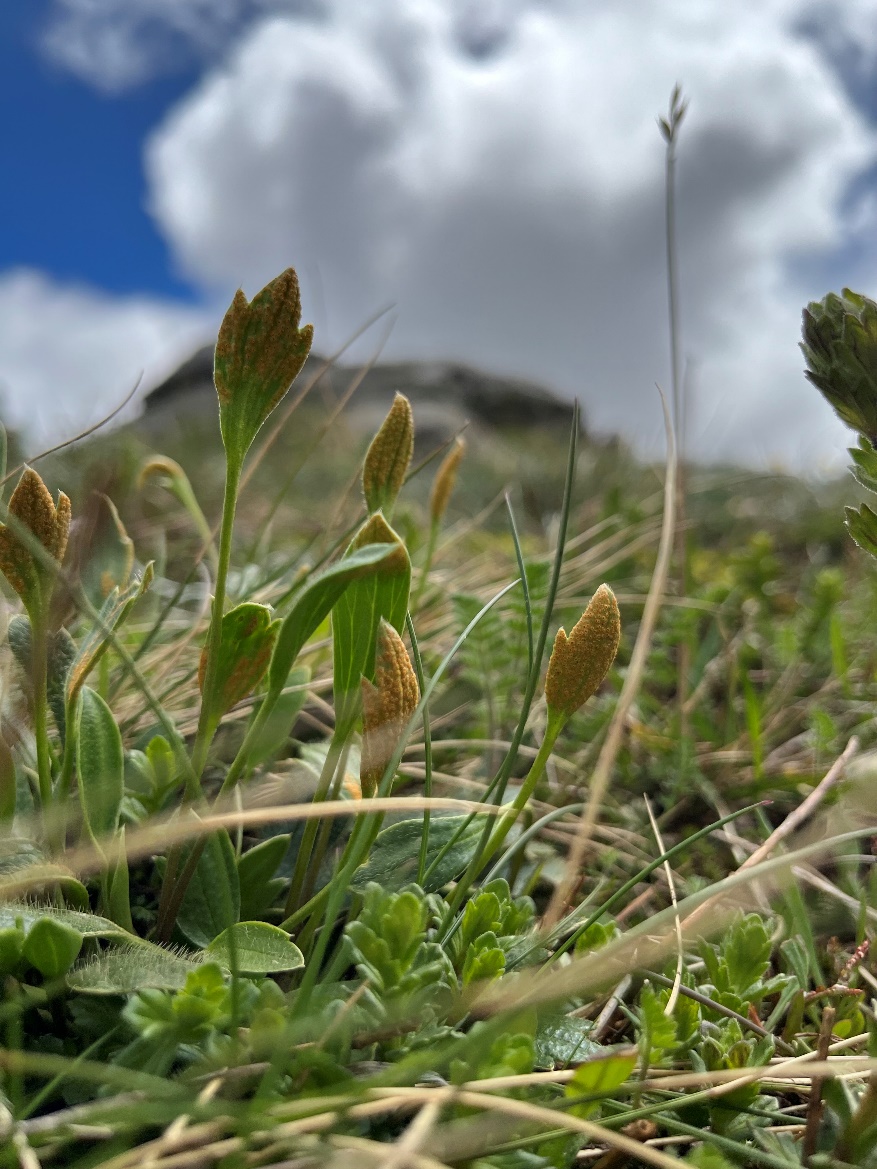 |

**Appendix S3.** Full statistical model results

*Table S3a. The results and values of models comparing the leaf damage (%) and the number of leaf damage types between different distribution positions of uphill, downhill and non-shifting species. The models are organised by the order of hypotheses (as described in the introduction). While to test our hypotheses, we were interested only in the interaction terms, this table includes all tested terms.*

|  | **Damage** | | | | | **Types** | | | | |
| --- | --- | --- | --- | --- | --- | --- | --- | --- | --- | --- |
|  | **value** | **SE** | **p-value** | **ANOVA**  **p-value** | **R^2^** | **value** | **SE** | **p-value** | **ANOVA**  **p-value** | **R^2^** |
| **Hypothesis 1: Leaf damage at the warm edge vs. core** | | | | | | | | | | |
| Intercept | 1.78 | 0.55 | <0.001 | <0.001 | Conditional: | -0.34 | 0.53 | 0.52 | 0.52 | Conditional: |
| Position- core | -0.13 | 0.48 | 0.78 | 0.78 | 0.43 | -0.16 | 0.26 | 0.54 | 0.54 | 0.49 |
| Shift direction - no | 0.95 | 0.69 | 0.17 | 0.26 | Marginal: | 0.48 | 0.67 | 0.48 | 0.42 | Marginal: |
| Shift direction - up | 0.18 | 0.65 | 0.78 |  | 0.07 | -0.25 | 0.64 | 0.69 |  | 0.06 |
| **Position - core * shift direction - no** | **-0.27** | **0.59** | **0.65** | **0.89** |  | **-0.06** | **0.32** | **0.85** | **0.81** |  |
| Position - core * shift direction - up | -0.25 | 0.55 | 0.65 |  |  | 0.09 | 0.30 | 0.76 |  |  |
| Individual: position: species | 0.40 |  |  |  |  | 0.08 |  |  |  |  |
| Position: species | 0.10 |  |  |  |  | 0.04 |  |  |  |  |
| Species | 0.32 |  |  |  |  | 0.48 |  |  |  |  |
| **Hypothesis 2: Leaf damage at the cold edge vs. core** | | | | | | | | | | |
| Intercept | 1.61 | 0.56 | 0.00 | 0.00 | Conditional: | -0.53 | 0.41 | 0.20 | 0.20 | Conditional: |
| Position – cold | 0.27 | 0.64 | 0.67 | 0.67 | 0.47 | 0.14 | 0.40 | 0.74 | 0.74 | 0.44 |
| Shift direction - no | 0.74 | 0.71 | 0.30 | 0.38 | Marginal: | 0.44 | 0.53 | 0.41 | 0.41 | Marginal: |
| Shift direction - up | -0.03 | 0.67 | 0.96 |  | 0.10 | -0.14 | 0.51 | 0.79 |  | 0.10 |
| **Position - cold * shift direction - no** | **-0.08** | **0.82** | **0.92** | **0.63** |  | **0.21** | **0.52** | **0.69** | **0.55** |  |
| Position - cold * shift direction - up | -0.61 | 0.78 | 0.43 |  |  | -0.25 | 0.49 | 0.60 |  |  |
| Individual: position: species | 0.35 |  |  |  |  | 0.13 |  |  |  |  |
| Position: species | 0.34 |  |  |  |  | 0.14 |  |  |  |  |
| Species | 0.20 |  |  |  |  | 0.18 |  |  |  |  |
| **Hypothesis 3: Leaf damage at the expanding edges vs. core** | | | | | | | | | | |
| Intercept | 1.61 | 0.64 | 0.01 | 0.01 | Conditional: | -0.53 | 0.53 | 0.32 | 0.32 | Conditional: |
| Edge type - expanding | 0.26 | 0.84 | 0.76 | 0.76 | 0.48 | 0.11 | 0.52 | 0.83 | 0.83 | 0.47 |
| shift direction - up | -0.04 | 0.78 | 0.96 | 0.96 | Marginal: | -0.16 | 0.65 | 0.80 | 0.80 | Marginal: |
| **edge type - expanding * shift direction - up** | **-0.60** | **0.99** | **0.54** | **0.54** | 0.02 | **-0.24** | **0.61** | **0.70** | **0.70** | 0.01 |
| Individual: position: species | 0.55 |  |  |  |  | 0.22 |  |  |  |  |
| Position: species | 0.50 |  |  |  |  | 0.19 |  |  |  |  |
| Species | 0.21 |  |  |  |  | 0.33 |  |  |  |  |
| **Hypothesis 4: Leaf damage across the range of non-shifting species** | | | | | | | | | | |
| Intercept | 2.75 | 0.36 | <0.001 | <0.001 | Conditional: | 0.14 | 0.20 | 0.48 | 0.47 | Conditional: |
| **position – core** | **-0.53** | **0.14** | **0.001** | **0.001** | 0.35 | **-0.28** | **0.07** | **<0.001** | **<0.001** | 0.22 |
| position - cold | -0.13 | 0.14 | 0.37 |  | Marginal: | 0.16 | 0.07 | 0.02 |  | Marginal: |
| Species: individual | 0.19 |  |  |  | 0.03 | 0.03 |  |  |  | 0.05 |
| Species | 0.35 |  |  |  |  | 0.11 |  |  |  |  |

**Appendix S4.** Statistical analysis and pairwise comparison of leaf damage (%) and the number of damage types between positions, for individual species.

*Table S4a. Results of the Generalised Linear Model analyses of leaf damage (%) of each species.*

| **species** | **term** | **Chisq** | **Df** | **Pr(>Chisq)** |
| --- | --- | --- | --- | --- |
| *Aciphylla glacialis* | (Intercept) | 157.53 | 1 | <0.001 |
| *Aciphylla glacialis* | Position | 8.74 | 2 | 0.01 |
| *Dichosciadium ranunculaceum* var. *ranunculaceum* | (Intercept) | 145.12 | 1 | <0.001 |
| *Dichosciadium ranunculaceum* var*. ranunculaceum* | Position | 87.93 | 2 | <0.001 |
| *Lycopodium fastigiatum* | (Intercept) | 44.85 | 1 | <0.001 |
| *Lycopodium fastigiatum* | Position | 0.70 | 2 | 0.71 |
| *Nematolepis ovatifolia* | (Intercept) | 1067.13 | 1 | <0.001 |
| *Nematolepis ovatifolia* | Position | 22.91 | 2 | <0.001 |
| *Orites lancifolius* | (Intercept) | 256.25 | 1 | <0.001 |
| *Orites lancifolius* | Position | 8.52 | 2 | 0.01 |
| *Pappochroma setosum* | (Intercept) | 8.24 | 1 | <0.001 |
| *Pappochroma setosum* | Position | 6.43 | 2 | 0.04 |
| *Pentachondra pumila* | (Intercept) | 99.64 | 1 | <0.001 |
| *Pentachondra pumila* | Position | 3.67 | 2 | 0.16 |
| *Prasophyllum tadgellianum* | (Intercept) | 53.56 | 1 | <0.001 |
| *Prasophyllum tadgellianum* | Position | 1.11 | 2 | 0.57 |

*Table S4b. Pairwise comparison of leaf damage (%) between the distribution positions of all species.*

| **species** | **contrast** | **ratio** | **SE** | **df** | **null** | **z.ratio** | **p.value** |
| --- | --- | --- | --- | --- | --- | --- | --- |
| *Aciphylla glacialis* | warm / core | 1.43 | 0.30 | Inf | 1 | 1.68 | 0.21 |
| *Aciphylla glacialis* | warm / cold | 0.77 | 0.16 | Inf | 1 | -1.24 | 0.43 |
| *Aciphylla glacialis* | core / cold | 0.54 | 0.11 | Inf | 1 | -2.94 | 0.01 |
| *Dichosciadium ranunculaceum* var*. ranunculaceum* | warm / core | 0.60 | 0.15 | Inf | 1 | -2.02 | 0.11 |
| *Dichosciadium ranunculaceum* var*. ranunculaceum* | warm / cold | 6.41 | 1.68 | Inf | 1 | 7.09 | <0.001 |
| *Dichosciadium ranunculaceum* var*. ranunculaceum* | core / cold | 10.60 | 2.78 | Inf | 1 | 9.01 | <0.001 |
| *Lycopodium fastigiatum* | warm / core | 0.91 | 0.28 | Inf | 1 | -0.32 | 0.94 |
| *Lycopodium fastigiatum* | warm / cold | 0.77 | 0.24 | Inf | 1 | -0.83 | 0.69 |
| *Lycopodium fastigiatum* | core / cold | 0.85 | 0.26 | Inf | 1 | -0.52 | 0.86 |
| *Nematolepis ovatifolia* | warm / core | 2.22 | 0.37 | Inf | 1 | 4.76 | <0.001 |
| *Nematolepis ovatifolia* | warm / cold | 1.56 | 0.26 | Inf | 1 | 2.72 | 0.02 |
| *Nematolepis ovatifolia* | core / cold | 0.70 | 0.12 | Inf | 1 | -2.08 | 0.09 |
| *Orites lancifolius* | warm / core | 1.31 | 0.27 | Inf | 1 | 1.29 | 0.40 |
| *Orites lancifolius* | warm / cold | 0.69 | 0.15 | Inf | 1 | -1.70 | 0.20 |
| *Orites lancifolius* | core / cold | 0.53 | 0.12 | Inf | 1 | -2.92 | 0.01 |
| *Pappochroma setosum* | warm / core | 4.19 | 2.37 | Inf | 1 | 2.53 | 0.03 |
| *Pappochroma setosum* | warm / cold | 2.13 | 1.16 | Inf | 1 | 1.38 | 0.35 |
| *Pappochroma setosum* | core / cold | 0.51 | 0.28 | Inf | 1 | -1.22 | 0.44 |
| *Pentachondra pumila* | warm / core | 1.37 | 0.42 | Inf | 1 | 1.02 | 0.56 |
| *Pentachondra pumila* | warm / cold | 1.81 | 0.56 | Inf | 1 | 1.91 | 0.13 |
| *Pentachondra pumila* | core / cold | 1.32 | 0.41 | Inf | 1 | 0.88 | 0.65 |
| *Prasophyllum tadgellianum* | warm / core | 0.79 | 0.34 | Inf | 1 | -0.54 | 0.85 |
| *Prasophyllum tadgellianum* | warm / cold | 1.26 | 0.54 | Inf | 1 | 0.53 | 0.86 |
| *Prasophyllum tadgellianum* | core / cold | 1.59 | 0.69 | Inf | 1 | 1.05 | 0.54 |

*Table S4c. Results of the Generalised Linear Model analyses of the number of damage types of each species.*

| **species** | **term** | **Chisq** | **Df** | **Pr(>Chisq)** |
| --- | --- | --- | --- | --- |
| *Aciphylla glacialis* | (Intercept) | 0.01 | 1 | 0.93 |
| *Aciphylla glacialis* | Position | 44.50 | 2 | <0.001 |
| *Dichosciadium ranunculaceum* var. *ranunculaceum* | (Intercept) | 6.85 | 1 | 0.01 |
| *Dichosciadium ranunculaceum* var. *ranunculaceum* | Position | 46.84 | 2 | <0.001 |
| *Lycopodium fastigiatum* | (Intercept) | 67.13 | 1 | <0.001 |
| *Lycopodium fastigiatum* | Position | 4.77 | 2 | 0.09 |
| *Nematolepis ovatifolia* | (Intercept) | 9.50 | 1 | <0.001 |
| *Nematolepis ovatifolia* | Position | 9.06 | 2 | 0.01 |
| *Orites lancifolius* | (Intercept) | 0.03 | 1 | 0.87 |
| *Orites lancifolius* | Position | 3.20 | 2 | 0.20 |
| *Pappochroma setosum* | (Intercept) | 58.98 | 1 | <0.001 |
| *Pappochroma setosum* | Position | 15.70 | 2 | <0.001 |
| *Pentachondra pumila* | (Intercept) | 126.03 | 1 | <0.001 |
| *Pentachondra pumila* | Position | 15.59 | 2 | <0.001 |
| *Prasophyllum tadgellianum* | (Intercept) | 24.55 | 1 | <0.001 |
| *Prasophyllum tadgellianum* | Position | 0.14 | 2 | 0.93 |

*Table S4d. Pairwise comparison of the number of damage types between the distribution positions of all species.*

| **species** | **contrast** | **ratio** | **SE** | **Df** | **null** | **z.ratio** | **p.value** |
| --- | --- | --- | --- | --- | --- | --- | --- |
| Aciphylla glacialis | warm/core | 1.48 | 0.16 | Inf | 1 | 3.62 | <0.001 |
| *Aciphylla glacialis* | warm/cold | 0.73 | 0.08 | Inf | 1 | -2.89 | 0.01 |
| *Aciphylla glacialis* | core/cold | 0.50 | 0.05 | Inf | 1 | -6.66 | <0.001 |
| *Dichosciadium ranunculaceum* var. *ranunculaceum* | warm/core | 1.44 | 0.28 | Inf | 1 | 1.86 | 0.15 |
| *Dichosciadium ranunculaceum* var. *ranunculaceum* | warm/cold | 3.93 | 0.81 | Inf | 1 | 6.69 | <0.001 |
| *Dichosciadium ranunculaceum* var. *ranunculaceum* | core/cold | 2.73 | 0.56 | Inf | 1 | 4.87 | <0.001 |
| *Lycopodium fastigiatum* | warm/core | 1.01 | 0.23 | Inf | 1 | 0.06 | 1.00 |
| *Lycopodium fastigiatum* | warm/cold | 0.66 | 0.15 | Inf | 1 | -1.85 | 0.15 |
| *Lycopodium fastigiatum* | core/cold | 0.66 | 0.15 | Inf | 1 | -1.90 | 0.14 |
| *Nematolepis ovatifolia* | warm/core | 1.20 | 0.09 | Inf | 1 | 2.56 | 0.03 |
| *Nematolepis ovatifolia* | warm/cold | 0.99 | 0.07 | Inf | 1 | -0.17 | 0.98 |
| *Nematolepis ovatifolia* | core/cold | 0.82 | 0.06 | Inf | 1 | -2.73 | 0.02 |
| *Orites lancifolius* | warm/core | 0.92 | 0.25 | Inf | 1 | -0.29 | 0.95 |
| *Orites lancifolius* | warm/cold | 1.47 | 0.40 | Inf | 1 | 1.40 | 0.34 |
| *Orites lancifolius* | core/cold | 1.59 | 0.44 | Inf | 1 | 1.69 | 0.21 |
| *Pappochroma setosum* | warm/core | 1.75 | 0.54 | Inf | 1 | 1.82 | 0.16 |
| *Pappochroma setosum* | warm/cold | 0.54 | 0.16 | Inf | 1 | -2.14 | 0.08 |
| *Pappochroma setosum* | core/cold | 0.31 | 0.09 | Inf | 1 | -3.94 | <0.001 |
| *Pentachondra pumila* | warm/core | 0.57 | 0.08 | Inf | 1 | -3.91 | <0.001 |
| *Pentachondra pumila* | warm/cold | 0.68 | 0.10 | Inf | 1 | -2.65 | 0.02 |
| *Pentachondra pumila* | core/cold | 1.19 | 0.16 | Inf | 1 | 1.28 | 0.40 |
| *Prasophyllum tadgellianum* | warm/core | 1.00 | 0.20 | Inf | 1 | -0.01 | 1.00 |
| *Prasophyllum tadgellianum* | warm/cold | 0.94 | 0.17 | Inf | 1 | -0.34 | 0.94 |
| *Prasophyllum tadgellianum* | core/cold | 0.94 | 0.19 | Inf | 1 | -0.29 | 0.95 |

**Appendix S5.** Summary of the mean leaf damage values across the distribution position of the sampled species

*Table S5a. The mean amount of damage (%) and number of damage types across the distribution positions of the sampled species.*

| **Species** | **Position** | **Mean leaf damage (%)** | **Mean damage types** |
| --- | --- | --- | --- |
| *Aciphylla glacialis* | Warm | 7.80 | 1.01 |
| *Aciphylla glacialis* | Core | 4.67 | 0.71 |
| *Aciphylla glacialis* | Core | 5.36 | 0.68 |
| *Aciphylla glacialis* | Cold | 9.81 | 1.37 |
| *Dichosciadium ranunculaceum* var*. ranunculaceum* | Warm | 9.63 | 1.51 |
| *Dichosciadium ranunculaceum* var*. ranunculaceum* | Core | 14.84 | 1.09 |
| *Dichosciadium ranunculaceum* var*. ranunculaceum* | Cold | 1.67 | 0.40 |
| *Lycopodium fastigiatum* | Warm | 5.87 | 0.29 |
| *Lycopodium fastigiatum* | Core | 5.37 | 0.27 |
| *Lycopodium fastigiatum* | Cold | 6.11 | 0.44 |
| *Nematolepis ovatifolia* | Warm | 42.36 | 0.86 |
| *Nematolepis ovatifolia* | Core | 20.82 | 0.72 |
| *Nematolepis ovatifolia* | Cold | 27.64 | 0.87 |
| *Orites lancifolius* | Warm | 11.03 | 0.99 |
| *Orites lancifolius* | Core | 8.63 | 1.08 |
| *Orites lancifolius* | Cold | 17.25 | 0.87 |
| *Pappochroma setosum* | Warm | 6.26 | 0.24 |
| *Pappochroma setosum* | Core | 0.96 | 0.13 |
| *Pappochroma setosum* | Cold | 2.61 | 0.40 |
| *Pentachondra pumila* | Warm | 9.23 | 0.31 |
| *Pentachondra pumila* | Core | 7.61 | 0.53 |
| *Pentachondra pumila* | Cold | 5.36 | 0.45 |
| *Prasophyllum tadgellianum* | Warm | 12.08 | 1.82 |
| *Prasophyllum tadgellianum* | Core | 16.55 | 1.82 |
| *Prasophyllum tadgellianum* | Cold | 8.19 | 1.93 |
| *Ranunculus anemoneus* | Warm | 15.42 | 2.15 |
| *Ranunculus anemoneus* | Core | 5.32 | 1.38 |
| *Ranunculus anemoneus* | Cold | 8.75 | 1.21 |

*Figure S5b. (a) The mean leaf damage (%) and (b) the mean number of damage types across elevation. The circles represent the mean leaf damage (a) and the number of damage types (b) sampled at one distribution position of each species; the dashed lines connect the three distribution positions of each species. The error bars span ± Standard Error.*


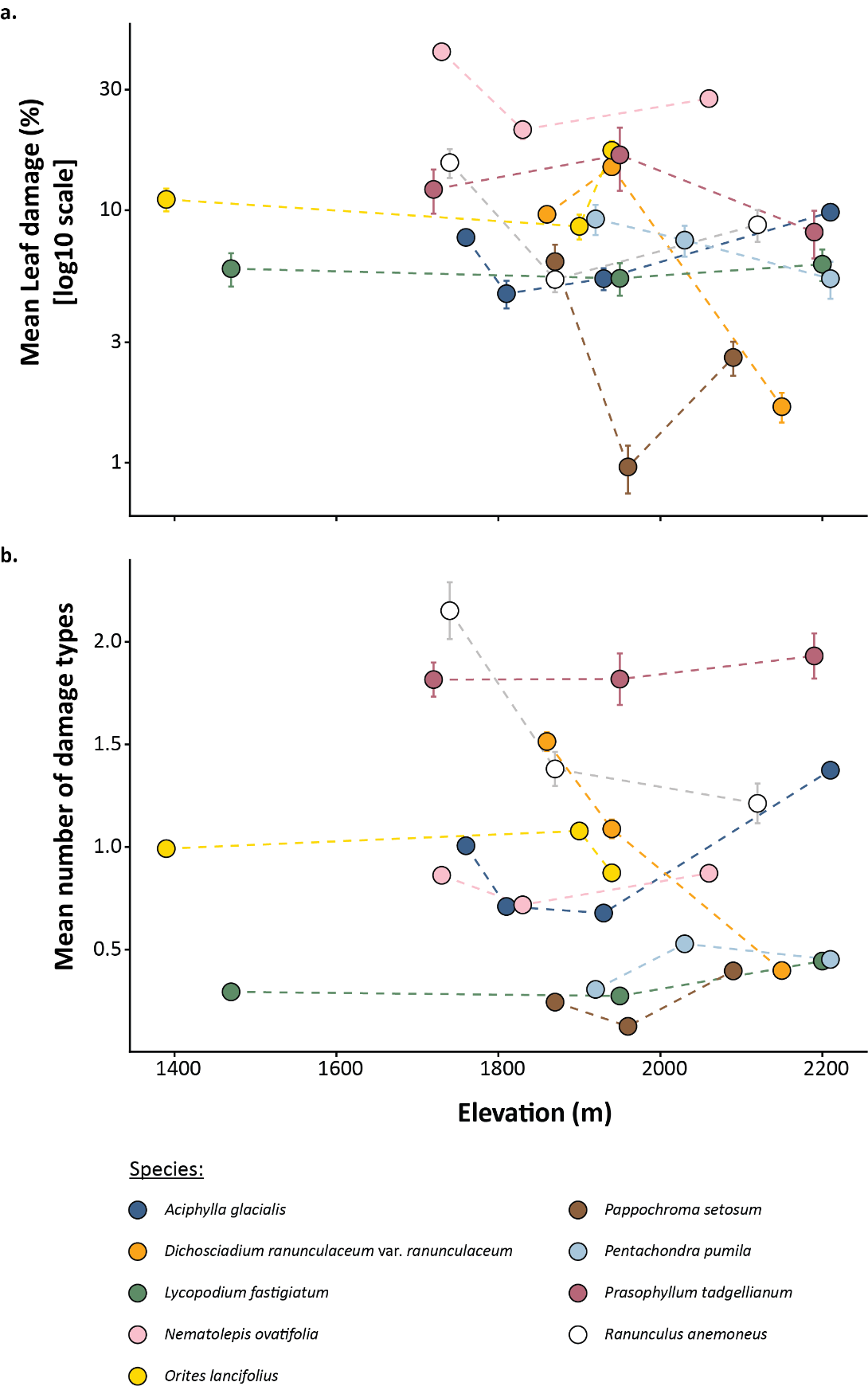

Supplement: Supplementary file 1 — Appendix S1: A list of all sampled locations per species, their coordinates and altitude, and the number of sampled individuals. Appendix S2: The types of damage recorded during the survey. Appendix S3: Full statistical model results. Appendix S4: Statistical analysis and pairwise comparison of leaf damage (%) and the number of damage types between positions, for individual species. Appendix S5: Summary of the mean leaf damage values across the distribution position of the sampled species. [file ECE3-16-e73437-s001.docx]
